# 2-Amino-6-nitro-1,3-benzo­thia­zol-3-ium 3-carb­oxy-4-hy­droxy­benzene-1-sulfonate

**DOI:** 10.1107/S241431462500478X

**Published:** 2025-05-30

**Authors:** Joseph Tsemeugne, Didier Forest Kouganou Djossu, Dieter Schollmeyer, Pierre Mkounga

**Affiliations:** a Laboratory of Natural Products and Applied Organic Synthesis (LANAPOS), Department of Organic Chemistry, Faculty of Science, University of Yaounde I, PO Box 812 Yaounde, Republic of , Cameroon; b Department of Chemistry, University of Mainz, Duesbergweg 10-14, 55099 Mainz, Germany; University of Aberdeen, United Kingdom

**Keywords:** crystal structure, salt, hydrogen bonding

## Abstract

In the title salt, C_7_H_6_N_3_O_2_S^+^·C_7_H_5_O_6_S^−^, the cation is protonated at the thia­zole N atom and the dihedral angle between the nitro group and its attached benzene ring is 3.6 (4)°. In the anion, the sulfonate group is deprotonated and the dihedral angle between the carb­oxy­lic acid grouping and its attached ring is 7.1 (4)° and an intra­molecular O—H⋯O hydrogen bond occurs. In the crystal, cation-to-anion N—H⋯O and anion-to-anion O—H⋯O hydrogen bonds link the component ions into (101) sheets. Aromatic π–π stacking and weak C—H⋯O and C—H⋯S inter­actions also occur.

## Abstract 2

In the title salt, the cation is protonated at the thia­zole N atom and in the anion, the sulfonate group is deprotonated and an intra­molecular O—H⋯O hydrogen bond occurs. In the crystal, cation-to-anion N—H⋯O and anion-to-anion O—H⋯O hydrogen bonds link the component ions into (101) sheets.

## Graphical abstract

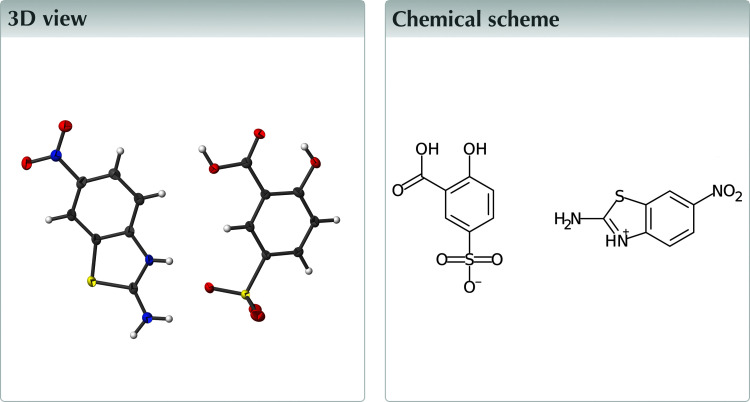

## Structure description

Salicylic acid and benzo­thia­zole derivatives are present in several skeletons with different biological activities (Pavle *et al.*, 2015 ▸; Ekinci *et al.*, 2011 ▸; Yadav *et al.*, 2023 ▸; Djuidje *et al.*, 2022 ▸). The assembly of several pharmacophores within a single skeleton, known as hybrid mol­ecules (Shaveta *et al.*, 2016 ▸), is an emerging approach to drug design. The association of pharmacophores into one mol­ecule can be achieved through the creation of strong σ bonds or by the formation of ionic bonds through the synthesis of salts (Singh *et al.*, 2022 ▸). The formulation of drugs in salt form has the advantage of improving the solubility of the active ingredient in the physiological fluids (Gupta *et al.*, 2018 ▸), as well as solving problems of stability, toxicity, and low absorption (Sekhon, 2011 ▸). Salts of benzo­thia­zole derivatives possess auxin-like activity (Giannella *et al.*, 1971 ▸), while salts of salicylic acid are used in the flavoring of foods, sweets, beverages and pharmaceuticals (Ekinci *et al.*, 2011 ▸).

**Figure 1:**
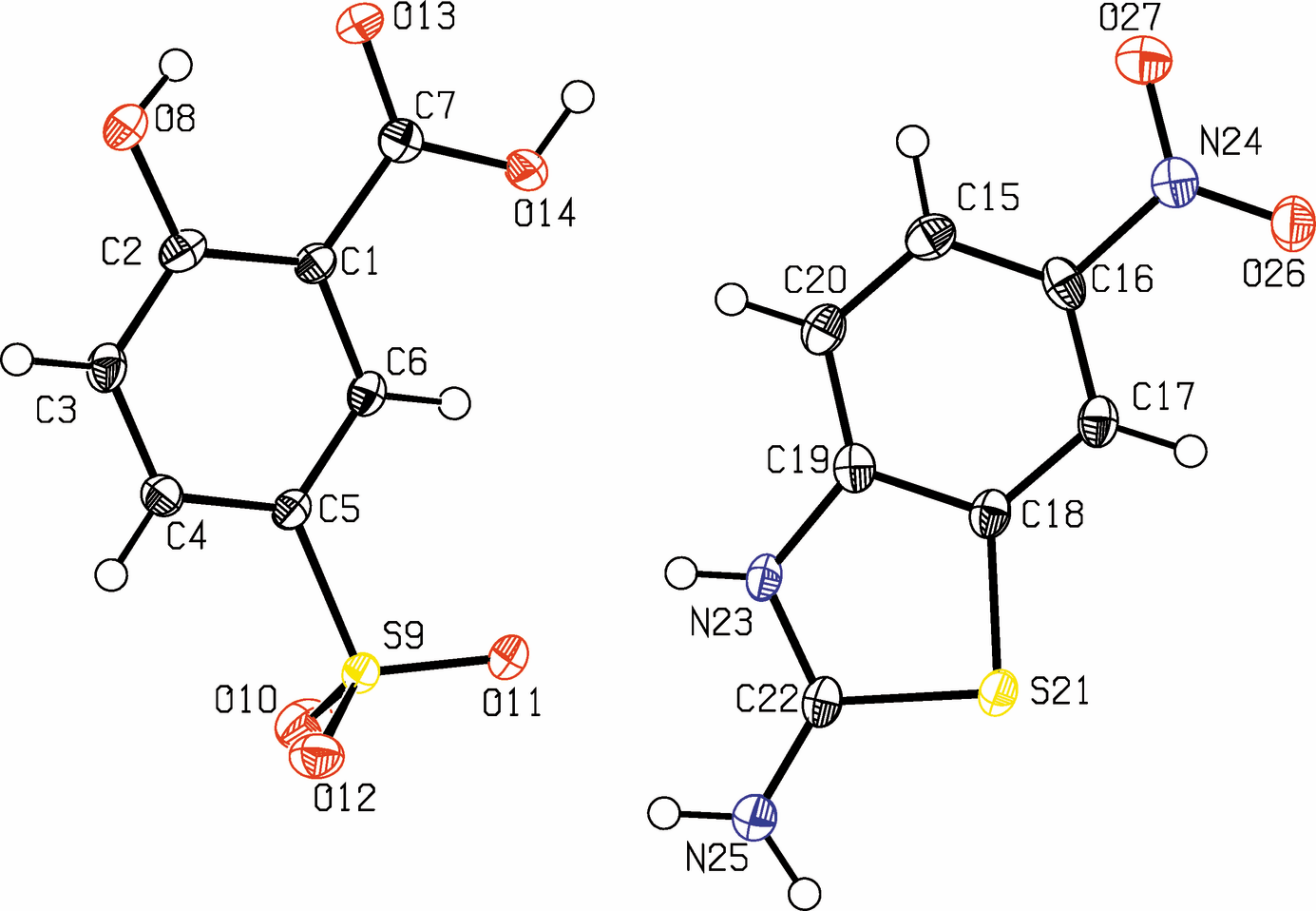
The asymmetric unit of the title compound with displacement ellipsoids drawn at the 50% probability level.

The combination of salicylic acid and 2-amino­benzo­thia­zole fragments could lead to a hybrid salt that combines the biological potentials of the different fragments and we now describe the structure of the title mol­ecular salt, C_7_H_6_N_3_O_2_S^+^·C_7_H_5_O_6_S^−^ (Fig. 1 ▸). The cation is protonated at the thia­zole N23 atom and the dihedral angle between the N24/O26/O27 nitro group and its attached C15–C20 benzene ring is 3.6 (4)°. In the anion, the S9/O10/O11/O12 sulfonate group is deprotonated and the dihedral angle between the C7/O13/O14 carb­oxy­lic acid grouping and its attached C1–C6 ring is 7.1 (2)°. An intra­molecular O8—H8⋯O13 hydrogen bond (Table 1 ▸) occurs in the anion. The cation and anion in the asymmetric unit lie in approximately the same plane with a dihedral angle between the best planes through the ring systems of 10.75 (12)°.

**Figure 2:**
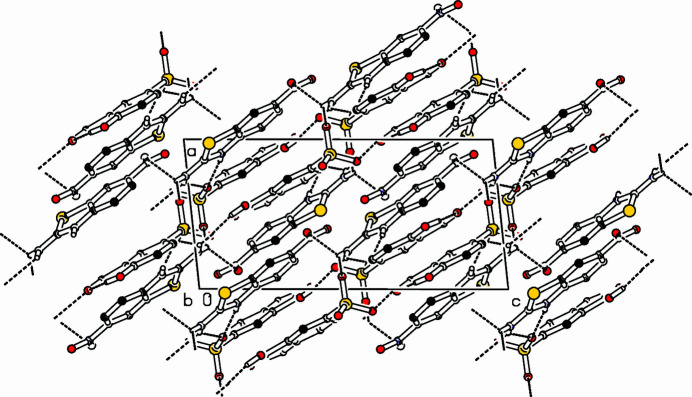
Part of the packing diagram viewed along *b*-axis direction. Hydrogen bonds are shown with dashed lines. Hydrogen atoms attached to carbon atoms are omitted for clarity.

In the crystal (Fig. 2 ▸), anion-to-anion O14—H144⋯O12 hydrogen bonds link the anions into [10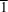] chains and cation-to-anion N—H⋯O links generate (10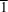) sheets. Aromatic π–π stacking with a centroid–centroid distance of 3.7043 (18) Å and a slippage of 0.862 Å occurs between the centroids of the C1–C6 and C15–C20 benzene rings and weak C—H⋯O and C—H⋯S inter­actions also occur.

## Synthesis and crystallization

A mixture of 5 ml of an ethano­lic solution of 2-amino-6-nitro­benzo­thia­zole (0.975 g, 5 mmol) and 5 ml of an ethano­lic solution of sulfosalicylic acid (1.225 g, 5 mmol) was refluxed for 2 h. The yellow crystalline precipitate was collected on a filter and recrystallized from ethanol solution to give 1.951 g (98%) of the title salt in the form of yellow blocks. For spectroscopic and analytical details, see Djossu *et al.* (2025 ▸).

## Refinement

Crystal data, data collection and structure refinement details are summarized in Table 2 ▸.

## Supplementary Material

Crystal structure: contains datablock(s) I, global. DOI: 10.1107/S241431462500478X/hb4518sup1.cif

Structure factors: contains datablock(s) I. DOI: 10.1107/S241431462500478X/hb4518Isup2.hkl

Supporting information file. DOI: 10.1107/S241431462500478X/hb4518Isup3.cml

CCDC reference: 2431842

Additional supporting information:  crystallographic information; 3D view; checkCIF report

Additional supporting information:  crystallographic information; 3D view; checkCIF report

## Figures and Tables

**Table 1:** Hydrogen-bond geometry (Å, °)

*D*—H⋯*A*	*D*—H	H⋯*A*	*D*⋯*A*	*D*—H⋯*A*
O8—H8⋯O13	0.81 (5)	1.94 (5)	2.631 (4)	143 (5)
O14—H144⋯O12^i^	0.87 (5)	1.70 (5)	2.564 (3)	168 (5)
N23—H23⋯O11	0.82 (5)	1.95 (5)	2.682 (4)	150 (4)
N25—H25*A*⋯O11	0.89 (5)	2.20 (5)	2.920 (4)	138 (4)
N25—H25*A*⋯O13^ii^	0.89 (5)	2.42 (5)	2.964 (4)	119 (4)
N25—H25*B*⋯O10^iii^	0.90 (5)	2.19 (5)	2.967 (4)	144 (4)
N25—H25*B*⋯O26^iv^	0.90 (5)	2.51 (5)	3.177 (4)	132 (4)
C3—H3⋯S21^v^	0.95	2.85	3.670 (4)	145
C6—H6⋯O8^vi^	0.95	2.60	3.446 (4)	149
C20—H20⋯O14	0.95	2.50	3.413 (4)	160

Symmetry codes: (i) 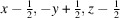; (ii) 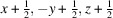; (iii) 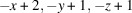; (iv) 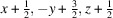; (v) 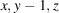; (vi) 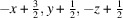.

**Table 2:** Experimental details

Crystal data	
Chemical formula	C_7_H_6_N_3_O_2_S^+^·C_7_H_5_O_6_S^−^
*M*_r_	413.38
Crystal system, space group	Monoclinic, *P*2_1_/*n*
Temperature (K)	120
*a*, *b*, *c* (Å)	7.8421 (3), 12.3037 (5), 16.0821 (6)
β (°)	96.081 (3)
*V* (Å^3^)	1542.98 (10)
*Z*	4
Radiation type	Mo *K*α
μ (mm^−1^)	0.40
Crystal size (mm)	0.15 × 0.08 × 0.05

Data collection	
Diffractometer	Stoe *IPDS* 2T
Absorption correction	Integration (*X-RED*; Stoe & Cie, 2020 ▸)
*T*_min_, *T*_max_	0.952, 0.977
No. of measured, independent and observed [*I* > 2σ(*I*)] reflections	7249, 3652, 2894
*R*_int_	0.036
(sin θ/λ)_max_ (Å^−1^)	0.659

Refinement	
*R*[*F*^2^ > 2σ(*F*^2^)], *wR*(*F*^2^), *S*	0.060, 0.147, 1.16
No. of reflections	3652
No. of parameters	259
H-atom treatment	H atoms treated by a mixture of independent and constrained refinement
Δρ_max_, Δρ_min_ (e Å^−3^)	0.52, −0.54

Computer programs: *X-AREA WinXpose*, *Recipe* and *Integrate* (Stoe & Cie, 2020 ▸), *SHELXT2014* (Sheldrick, 2015*a* ▸), *SHELXL2019/2* (Sheldrick, 2015*b* ▸) and *PLATON* (Spek, 2009 ▸).

## full crystallographic data

### 2-Amino-6-nitro-1,3-benzothiazol-3-ium 3-carboxy-4-hydroxybenzene-1-sulfonate Crystal data

**Table:**

C_7_H_6_N_3_O_2_S^+^·C_7_H_5_O_6_S^−^	*F*(000) = 848
*M**_r_* = 413.38	*D*_x_ = 1.779 Mg m^−^^3^
Monoclinic, *P*2_1_/*n*	Mo *K*α radiation, λ = 0.71073 Å
*a* = 7.8421 (3) Å	Cell parameters from 7727 reflections
*b* = 12.3037 (5) Å	θ = 2.8–28.4°
*c* = 16.0821 (6) Å	µ = 0.40 mm^−^^1^
β = 96.081 (3)°	*T* = 120 K
*V* = 1542.98 (10) Å^3^	Block, yellow
*Z* = 4	0.15 × 0.08 × 0.05 mm

### 2-Amino-6-nitro-1,3-benzothiazol-3-ium 3-carboxy-4-hydroxybenzene-1-sulfonate Data collection

**Table:**

Stoe IPDS 2T diffractometer	3652 independent reflections
Radiation source: sealed X-ray tube, 12x0.4mm long-fine focus	2894 reflections with *I* > 2σ(*I*)
Detector resolution: 6.67 pixels mm^-1^	*R*_int_ = 0.036
rotation method, ω scans	θ_max_ = 28.0°, θ_min_ = 2.8°
Absorption correction: integration (X-RED; Stoe & Cie, 2020)	*h* = −10→10
*T*_min_ = 0.952, *T*_max_ = 0.977	*k* = −16→14
7249 measured reflections	*l* = −21→19

### 2-Amino-6-nitro-1,3-benzothiazol-3-ium 3-carboxy-4-hydroxybenzene-1-sulfonate Refinement

**Table:**

Refinement on *F*^2^	Primary atom site location: dual
Least-squares matrix: full	Hydrogen site location: mixed
*R*[*F*^2^ > 2σ(*F*^2^)] = 0.060	H atoms treated by a mixture of independent and constrained refinement
*wR*(*F*^2^) = 0.147	*w* = 1/[σ^2^(*F*_o_^2^) + (0.0372*P*)^2^ + 5.2258*P*] where *P* = (*F*_o_^2^ + 2*F*_c_^2^)/3
*S* = 1.16	(Δ/σ)_max_ < 0.001
3652 reflections	Δρ_max_ = 0.52 e Å^−^^3^
259 parameters	Δρ_min_ = −0.54 e Å^−^^3^
0 restraints	

### 2-Amino-6-nitro-1,3-benzothiazol-3-ium 3-carboxy-4-hydroxybenzene-1-sulfonate Special details

**Table:**

Geometry. All esds (except the esd in the dihedral angle between two l.s. planes) are estimated using the full covariance matrix. The cell esds are taken into account individually in the estimation of esds in distances, angles and torsion angles; correlations between esds in cell parameters are only used when they are defined by crystal symmetry. An approximate (isotropic) treatment of cell esds is used for estimating esds involving l.s. planes.
Refinement. Hydrogen atoms attached to carbon atoms were placed at calculated positions and were refined in the riding-model approximation with C—H = 0.99 Å and *U*_iso_(H) = 1.2 *U*_eq_(C).

### 2-Amino-6-nitro-1,3-benzothiazol-3-ium 3-carboxy-4-hydroxybenzene-1-sulfonate Fractional atomic coordinates and isotropic or equivalent isotropic displacement parameters (Å^2^)

**Table:**

	*x*	*y*	*z*	*U*_iso_*/*U*_eq_	
C1	0.6518 (4)	0.0838 (3)	0.2698 (2)	0.0158 (6)	
C2	0.6533 (4)	−0.0250 (3)	0.2968 (2)	0.0192 (7)	
C3	0.7293 (4)	−0.0523 (3)	0.3765 (2)	0.0195 (7)	
H3	0.731183	−0.125786	0.394662	0.023*	
C4	0.8015 (4)	0.0278 (3)	0.4288 (2)	0.0182 (7)	
H4	0.849399	0.009402	0.483748	0.022*	
C5	0.8050 (4)	0.1353 (3)	0.4021 (2)	0.0159 (6)	
C6	0.7324 (4)	0.1627 (3)	0.3226 (2)	0.0166 (6)	
H6	0.737463	0.235728	0.303905	0.020*	
C7	0.5593 (4)	0.1147 (3)	0.1885 (2)	0.0178 (6)	
O8	0.5814 (3)	−0.1059 (2)	0.24785 (17)	0.0249 (6)	
H8	0.544 (6)	−0.083 (4)	0.202 (3)	0.037*	
S9	0.89932 (10)	0.23678 (7)	0.46895 (5)	0.01698 (19)	
O10	1.0829 (3)	0.2334 (2)	0.46593 (17)	0.0284 (6)	
O11	0.8229 (3)	0.3393 (2)	0.44040 (16)	0.0271 (6)	
O12	0.8540 (3)	0.2077 (2)	0.55230 (15)	0.0253 (6)	
O13	0.4885 (3)	0.0483 (2)	0.13908 (15)	0.0218 (5)	
O14	0.5567 (3)	0.2203 (2)	0.17555 (15)	0.0225 (5)	
H144	0.494 (6)	0.238 (4)	0.129 (3)	0.034*	
C15	0.3114 (4)	0.5144 (3)	0.1841 (2)	0.0215 (7)	
H15	0.266975	0.475166	0.135708	0.026*	
C16	0.2677 (4)	0.6222 (3)	0.1932 (2)	0.0201 (7)	
C17	0.3252 (4)	0.6844 (3)	0.2627 (2)	0.0197 (7)	
H17	0.293611	0.758544	0.267279	0.024*	
C18	0.4311 (4)	0.6325 (3)	0.3250 (2)	0.0186 (7)	
C19	0.4790 (4)	0.5237 (3)	0.3160 (2)	0.0178 (6)	
C20	0.4201 (4)	0.4638 (3)	0.2456 (2)	0.0206 (7)	
H20	0.453567	0.390245	0.239810	0.025*	
S21	0.52666 (11)	0.68751 (7)	0.41888 (6)	0.0212 (2)	
C22	0.6366 (4)	0.5670 (3)	0.4408 (2)	0.0178 (6)	
N23	0.5936 (4)	0.4905 (2)	0.38293 (18)	0.0174 (6)	
H23	0.639 (5)	0.431 (4)	0.389 (3)	0.026*	
N24	0.1552 (4)	0.6738 (2)	0.12574 (18)	0.0210 (6)	
N25	0.7462 (4)	0.5524 (3)	0.50724 (19)	0.0223 (6)	
H25A	0.793 (6)	0.487 (4)	0.515 (3)	0.033*	
H25B	0.779 (6)	0.612 (4)	0.537 (3)	0.033*	
O26	0.1226 (3)	0.7708 (2)	0.13176 (16)	0.0264 (6)	
O27	0.0987 (3)	0.6171 (2)	0.06582 (16)	0.0267 (6)	

### 2-Amino-6-nitro-1,3-benzothiazol-3-ium 3-carboxy-4-hydroxybenzene-1-sulfonate Atomic displacement parameters (Å^2^)

**Table:**

	*U*^11^	*U*^22^	*U*^33^	*U*^12^	*U*^13^	*U*^23^
C1	0.0164 (14)	0.0157 (15)	0.0157 (14)	−0.0001 (12)	0.0034 (11)	−0.0020 (12)
C2	0.0161 (15)	0.0190 (17)	0.0228 (16)	−0.0018 (12)	0.0035 (12)	−0.0061 (13)
C3	0.0197 (16)	0.0137 (15)	0.0252 (17)	−0.0016 (12)	0.0027 (13)	0.0022 (13)
C4	0.0187 (15)	0.0192 (17)	0.0165 (15)	0.0022 (12)	0.0007 (12)	−0.0002 (12)
C5	0.0168 (15)	0.0133 (15)	0.0177 (15)	−0.0004 (11)	0.0020 (12)	−0.0020 (12)
C6	0.0169 (15)	0.0131 (15)	0.0202 (15)	−0.0008 (12)	0.0036 (12)	−0.0001 (12)
C7	0.0162 (15)	0.0206 (17)	0.0171 (15)	0.0012 (12)	0.0044 (12)	−0.0009 (13)
O8	0.0319 (14)	0.0145 (12)	0.0270 (13)	−0.0026 (10)	−0.0033 (11)	−0.0019 (10)
S9	0.0175 (4)	0.0133 (4)	0.0195 (4)	0.0008 (3)	−0.0009 (3)	−0.0015 (3)
O10	0.0186 (12)	0.0311 (15)	0.0353 (14)	−0.0030 (11)	0.0022 (10)	−0.0080 (12)
O11	0.0351 (14)	0.0142 (12)	0.0294 (14)	0.0026 (10)	−0.0090 (11)	−0.0037 (10)
O12	0.0298 (14)	0.0267 (14)	0.0189 (12)	−0.0043 (11)	0.0009 (10)	−0.0006 (10)
O13	0.0251 (13)	0.0188 (12)	0.0207 (12)	−0.0002 (10)	−0.0009 (10)	−0.0058 (10)
O14	0.0307 (13)	0.0174 (12)	0.0177 (11)	−0.0008 (10)	−0.0052 (10)	0.0026 (9)
C15	0.0224 (17)	0.0212 (18)	0.0214 (16)	−0.0033 (13)	0.0048 (13)	−0.0024 (13)
C16	0.0167 (15)	0.0218 (17)	0.0222 (16)	−0.0003 (13)	0.0036 (12)	0.0093 (13)
C17	0.0185 (15)	0.0155 (16)	0.0255 (17)	0.0000 (12)	0.0047 (13)	0.0027 (13)
C18	0.0159 (15)	0.0151 (16)	0.0251 (17)	−0.0012 (12)	0.0042 (12)	0.0007 (13)
C19	0.0159 (15)	0.0172 (16)	0.0211 (16)	−0.0006 (12)	0.0055 (12)	0.0032 (12)
C20	0.0219 (16)	0.0163 (16)	0.0244 (17)	−0.0028 (13)	0.0065 (13)	0.0008 (13)
S21	0.0220 (4)	0.0147 (4)	0.0261 (4)	0.0025 (3)	−0.0006 (3)	−0.0024 (3)
C22	0.0168 (15)	0.0143 (16)	0.0234 (16)	−0.0008 (12)	0.0072 (12)	0.0021 (13)
N23	0.0167 (13)	0.0122 (13)	0.0234 (14)	0.0018 (10)	0.0028 (11)	−0.0002 (11)
N24	0.0197 (14)	0.0214 (15)	0.0226 (14)	−0.0009 (11)	0.0049 (11)	0.0023 (12)
N25	0.0228 (15)	0.0196 (15)	0.0240 (15)	0.0018 (12)	−0.0002 (12)	−0.0015 (12)
O26	0.0306 (14)	0.0211 (13)	0.0277 (13)	0.0056 (11)	0.0037 (11)	0.0016 (10)
O27	0.0275 (13)	0.0300 (15)	0.0225 (12)	−0.0020 (11)	0.0020 (10)	−0.0013 (11)

### 2-Amino-6-nitro-1,3-benzothiazol-3-ium 3-carboxy-4-hydroxybenzene-1-sulfonate Geometric parameters (Å, º)

**Table:**

C1—C6	1.397 (4)	C15—C20	1.384 (5)
C1—C2	1.407 (5)	C15—H15	0.9500
C1—C7	1.477 (4)	C16—C17	1.389 (5)
C2—O8	1.354 (4)	C16—N24	1.468 (4)
C2—C3	1.396 (5)	C17—C18	1.388 (5)
C3—C4	1.377 (5)	C17—H17	0.9500
C3—H3	0.9500	C18—C19	1.402 (5)
C4—C5	1.393 (5)	C18—S21	1.748 (4)
C4—H4	0.9500	C19—N23	1.388 (4)
C5—C6	1.384 (4)	C19—C20	1.389 (5)
C5—S9	1.759 (3)	C20—H20	0.9500
C6—H6	0.9500	S21—C22	1.732 (3)
C7—O13	1.230 (4)	C22—N25	1.312 (5)
C7—O14	1.316 (4)	C22—N23	1.341 (4)
O8—H8	0.81 (5)	N23—H23	0.82 (5)
S9—O10	1.446 (3)	N24—O26	1.226 (4)
S9—O11	1.450 (3)	N24—O27	1.233 (4)
S9—O12	1.467 (3)	N25—H25A	0.89 (5)
O14—H144	0.87 (5)	N25—H25B	0.90 (5)
C15—C16	1.382 (5)		
			
C6—C1—C2	119.0 (3)	C16—C15—H15	120.1
C6—C1—C7	120.5 (3)	C20—C15—H15	120.1
C2—C1—C7	120.4 (3)	C15—C16—C17	123.5 (3)
O8—C2—C3	117.9 (3)	C15—C16—N24	118.2 (3)
O8—C2—C1	122.0 (3)	C17—C16—N24	118.3 (3)
C3—C2—C1	120.0 (3)	C18—C17—C16	116.5 (3)
C4—C3—C2	119.8 (3)	C18—C17—H17	121.8
C4—C3—H3	120.1	C16—C17—H17	121.8
C2—C3—H3	120.1	C17—C18—C19	120.7 (3)
C3—C4—C5	120.7 (3)	C17—C18—S21	127.9 (3)
C3—C4—H4	119.6	C19—C18—S21	111.3 (3)
C5—C4—H4	119.6	N23—C19—C20	127.4 (3)
C6—C5—C4	119.8 (3)	N23—C19—C18	111.1 (3)
C6—C5—S9	119.8 (3)	C20—C19—C18	121.4 (3)
C4—C5—S9	120.4 (2)	C15—C20—C19	118.1 (3)
C5—C6—C1	120.5 (3)	C15—C20—H20	120.9
C5—C6—H6	119.7	C19—C20—H20	120.9
C1—C6—H6	119.7	C22—S21—C18	90.16 (16)
O13—C7—O14	123.7 (3)	N25—C22—N23	124.3 (3)
O13—C7—C1	123.1 (3)	N25—C22—S21	123.5 (3)
O14—C7—C1	113.1 (3)	N23—C22—S21	112.2 (3)
C2—O8—H8	111 (4)	C22—N23—C19	115.1 (3)
O10—S9—O11	113.18 (17)	C22—N23—H23	118 (3)
O10—S9—O12	111.24 (16)	C19—N23—H23	127 (3)
O11—S9—O12	111.68 (16)	O26—N24—O27	123.4 (3)
O10—S9—C5	108.34 (16)	O26—N24—C16	118.4 (3)
O11—S9—C5	106.91 (15)	O27—N24—C16	118.1 (3)
O12—S9—C5	105.01 (15)	C22—N25—H25A	118 (3)
C7—O14—H144	112 (3)	C22—N25—H25B	116 (3)
C16—C15—C20	119.8 (3)	H25A—N25—H25B	125 (4)
			
C6—C1—C2—O8	−178.3 (3)	C20—C15—C16—N24	178.6 (3)
C7—C1—C2—O8	4.6 (5)	C15—C16—C17—C18	−0.3 (5)
C6—C1—C2—C3	2.3 (5)	N24—C16—C17—C18	180.0 (3)
C7—C1—C2—C3	−174.9 (3)	C16—C17—C18—C19	1.4 (5)
O8—C2—C3—C4	−179.0 (3)	C16—C17—C18—S21	179.1 (3)
C1—C2—C3—C4	0.5 (5)	C17—C18—C19—N23	176.1 (3)
C2—C3—C4—C5	−2.3 (5)	S21—C18—C19—N23	−1.9 (3)
C3—C4—C5—C6	1.2 (5)	C17—C18—C19—C20	−1.2 (5)
C3—C4—C5—S9	−179.4 (3)	S21—C18—C19—C20	−179.2 (3)
C4—C5—C6—C1	1.6 (5)	C16—C15—C20—C19	1.4 (5)
S9—C5—C6—C1	−177.8 (2)	N23—C19—C20—C15	−177.1 (3)
C2—C1—C6—C5	−3.3 (5)	C18—C19—C20—C15	−0.3 (5)
C7—C1—C6—C5	173.9 (3)	C17—C18—S21—C22	−174.8 (3)
C6—C1—C7—O13	178.8 (3)	C19—C18—S21—C22	3.1 (3)
C2—C1—C7—O13	−4.1 (5)	C18—S21—C22—N25	177.7 (3)
C6—C1—C7—O14	−3.0 (4)	C18—S21—C22—N23	−3.6 (3)
C2—C1—C7—O14	174.2 (3)	N25—C22—N23—C19	−178.1 (3)
C6—C5—S9—O10	−99.3 (3)	S21—C22—N23—C19	3.3 (4)
C4—C5—S9—O10	81.3 (3)	C20—C19—N23—C22	176.2 (3)
C6—C5—S9—O11	23.0 (3)	C18—C19—N23—C22	−0.9 (4)
C4—C5—S9—O11	−156.4 (3)	C15—C16—N24—O26	−176.6 (3)
C6—C5—S9—O12	141.7 (3)	C17—C16—N24—O26	3.1 (4)
C4—C5—S9—O12	−37.6 (3)	C15—C16—N24—O27	3.3 (4)
C20—C15—C16—C17	−1.1 (5)	C17—C16—N24—O27	−177.0 (3)

### 2-Amino-6-nitro-1,3-benzothiazol-3-ium 3-carboxy-4-hydroxybenzene-1-sulfonate Hydrogen-bond geometry (Å, º)

**Table:**

*D*—H···*A*	*D*—H	H···*A*	*D*···*A*	*D*—H···*A*
O8—H8···O13	0.81 (5)	1.94 (5)	2.631 (4)	143 (5)
O14—H144···O12^i^	0.87 (5)	1.70 (5)	2.564 (3)	168 (5)
N23—H23···O11	0.82 (5)	1.95 (5)	2.682 (4)	150 (4)
N25—H25*A*···O11	0.89 (5)	2.20 (5)	2.920 (4)	138 (4)
N25—H25*A*···O13^ii^	0.89 (5)	2.42 (5)	2.964 (4)	119 (4)
N25—H25*B*···O10^iii^	0.90 (5)	2.19 (5)	2.967 (4)	144 (4)
N25—H25*B*···O26^iv^	0.90 (5)	2.51 (5)	3.177 (4)	132 (4)
C3—H3···S21^v^	0.95	2.85	3.670 (4)	145
C6—H6···O8^vi^	0.95	2.60	3.446 (4)	149
C20—H20···O14	0.95	2.50	3.413 (4)	160

Symmetry codes: (i) *x*−1/2, −*y*+1/2, *z*−1/2; (ii) *x*+1/2, −*y*+1/2, *z*+1/2; (iii) −*x*+2, −*y*+1, −*z*+1; (iv) *x*+1/2, −*y*+3/2, *z*+1/2; (v) *x*, *y*−1, *z*; (vi) −*x*+3/2, *y*+1/2, −*z*+1/2.
